# Understanding the Phenomenon of Copper Ions Release from Copper-Modified TFC Membranes: A Mathematical and Experimental Methodology Using Shrinking Core Model

**DOI:** 10.3390/nano10061130

**Published:** 2020-06-08

**Authors:** Rodrigo Quezada, Yurieth Quintero, José Cristian Salgado, Humberto Estay, Andreina García

**Affiliations:** 1 Advanced Mining Technology Center (AMTC), Universidad de Chile, Av Tupper 2007 (AMTC Building), Santiago 8370451, Chile; rodrigo.quezada.m@uchile.cl (R.Q.); yurieth.quintero@amtc.cl (Y.Q.); 2 Department of Chemical Engineering, Biotechnology and Materials, Faculty of Physical and Mathematical Sciences, Universidad de Chile, Beauchef 851, Santiago 8370456, Chile; jsalgado@ing.uchile.cl

**Keywords:** biofouling, reverse osmosis, TFC membranes, copper-based NPs, shrinking core model

## Abstract

Type of metal and metal-oxide NPs added to modify Thin-Film Composites Reverse Osmosis Membranes (TFC-RO) can alter their anti-biofouling properties by changing the dissolution process. The development of a mathematical model can facilitate the selection of these NPs. This work consists of a mathematical and experimental methodology to understand copper-based NPs dissolution of three copper species incorporated into TFC-RO membranes: Cu-NPs, CuO-NPs and Cu-Oligomer complexes formed in situ during the polymerization process. Biocidal capacity of copper species into the membrane was evaluated using colony forming unit method (CFU) over *E. coli*. In addition, copper ion release kinetics for both NPs and modified membranes were determined. A model based on the shrinking core model (SCM) was validated and applied to determine the limiting rate step in the dissolution process and simulate the ion release kinetics. Fitted curves reached a good adjustment with the experimental data, demonstrating the SCM can be applied to predict ion release process for copper-based NPs in suspension and the modified membranes. All membranes reached similar inhibition rate >50%, however, differences in the dissolution level of copper-based NPs in membrane were noted, suggesting a dual-type effect that defined the copper toxicity into the membrane, associated to the dissolution capacity and ROS production.

## 1. Introduction

Thin film composite (TFC) membranes are the best-known polymer membranes for reverse osmosis (RO) with applications in the desalination of seawater. TFC-RO membranes typically consist of an ultrathin polyamide (PA) layer obtained from the reaction between diamine in water phase and acyl chloride in organic phase. The reaction is carried out on a porous polysulfone (PS). TFC membranes are the most used membranes in the RO process because of their excellent water flux and solute rejection [1,2,3], but they are susceptible to fouling issues [4].

Organic and inorganic fouling are major concerns in the desalination by RO membrane process industry worldwide [5]. Biological fouling or biofouling is the most critical concern, accounting for more than 45% of all cases of membrane fouling [6,7]. Attached bacterial growth on the membrane surface forms a biofilm, which increases the pressure drop in the system by creating additional resistance in the feed channel [6,8]. Biofouling is particularly challenging due to rapid propagation on polymeric surfaces [6,7,8,9]. There are several methods and strategies for the control of biofouling. The most common strategies include the physical removal of bacteria from the feed water using pretreatments with microfiltration and ultrafiltration membranes or the inactivation of cellular metabolism using biocides [5]. In the previously mentioned, regeneration capacity of some biofilms makes traditional treatments such as chlorine or backwashing ineffective where repeated treatments are needed, increasing operational costs and limiting the lifetime of the membrane [6,10].

On the other hand, superficial modification of membranes with nanomaterials possessing biocidal capacity has become an attractive method to grant anti-biofouling capacity to the RO membranes [1,6,11,12,13].

Several studies oriented to the incorporation of inorganic and metallic nanoparticles (NPs) like Silver [11,14,15], Zinc oxide [16,17] or Titanium oxide [18,19] with antibacterial capacity in RO membranes have been developed. The incorporation of inorganic nanoparticles, with antimicrobial properties, has been reported as an effective route to enhance the antibiofouling capacity of the RO membranes [11,19,20,21,22,23,24,25]. In particular, studies on the incorporation of copper-based nanoparticles (copper-based NPs) show a great capacity to induce toxic effects in bacteria, fungi and algae without depleting its permeability and salt rejection [20,26,27,28]. B. Rodriguez et al. [22] incorporated a copper oligomer complex in TFC-RO membranes in situ during the interfacial polymerization process, obtaining improved biocidal capacity, which was associated with the dissolution and release of copper ions. The membrane obtained good performance as a desalinization membrane. Similar results were obtained for the incorporation of Cu-NPs and CuO-NPs in TFC-RO membranes during the interfacial polymerization, demonstrating the viability of this route to modify and grant anti-biofouling capacity to TFC-RO membranes [20,21,26,28,29]. However, the influence of the type and characteristics of the NP on the mechanism of killing and the duration of the toxic capacity when nanoparticles are incorporated in RO membranes remain unclarified [30,31].

The NPs toxicity in microorganisms is determined by different NPs properties, such as type, shape, size and surface characteristics [26,32,33]. Two main routes are proposed for toxic activity of NPs: direct contact killing with microorganism, and ion release by the dissolution of the nanoparticle [11,33]. Contact killing is associated with the interaction between NPs and biologic structures such as cell membranes, proteins and genetic material (DNA and RNA), which leads to an increase in the reactive oxygen species (ROS) and the oxidative stress of the cell, ultimately promoting apoptosis [32]. Besides, metallic NPs dissolve at a higher rate than the bulk species, where metallic ions are toxic above physiological levels [33]. For copper, these ions can dislocate trace metals in proteins, inactivating or degrading them. Furthermore, the redox cycle produced between Cu^1+^ and Cu^2+^ also leads to an increase in ROS, both intracellularly and outside the cell [33,34,35]. For both Cu and CuO-NPs, studies have remarked the effect of contact killing and ion release in the toxicity of copper, suggesting that both mechanisms are necessary to ensure an optimum killing [36].

In contrast, when the nanoparticles are embedded in a polymeric matrix, such as the PA layer in the TFC-RO membranes, ion-induced cell death is expected to be predominantly higher than contact-killing [11,26]. In this regard, a study of the bactericidal capacity and its relation with the ion release capacity from copper-modified TFC membranes is needed. Likewise, a comparison between the different copper species with variations in their ability to generate anti-biofouling properties on TFC membranes has not been made [21,22,28].

A useful way to describe a mechanism of action and to obtain details about the biocidal properties of copper-based NPs in TFC-RO membranes is the use of mathematical models. There are few studies that attempt to model some of the biocidal mechanisms of metallic nanoparticles and how the design parameters influence NP-toxicity. For ion dissolution of NPs, most of the studies show that the dissolution follows first-order kinetics, as suggested by Noyes-Whitney equation [37]. However, this equation does not include design parameters of the NP (e.g., size), so it cannot predict the effect of changes in these parameters in the dissolution. A few other studies have included one or more of the characteristics of NPs with the dissolution capacity in mathematical models. Zhang et al. [38] derived a model based on the sphere collision theory using Arrhenius equation to describe the dissolution of Ag-NPs, including the size and concentration of the nanoparticle as a parameter in the model. Both size and concentration have a direct impact on its dissolution rate. Small NPs have higher solubility due to higher specific surface area and higher enthalpies of formation compared to bulk species. Accordingly, a higher concentration of NPs increases the ion release rate. Kent et al. [39] developed a model to estimate the persistence of copper-based nanomaterials under saturated solutions. The study shows that the initial amount of copper in the surface influences the lifespan of the particles, associated with the initial radius of the NP. Nevertheless, none of these models consider the passivation in dissolution or the effect on the ions release due to the presence of a polymeric layer such as the polyamide layer in the TFC-RO membranes. Rate limiting step determination for the dissolution reaction is also not considered.

**Figure 1:**
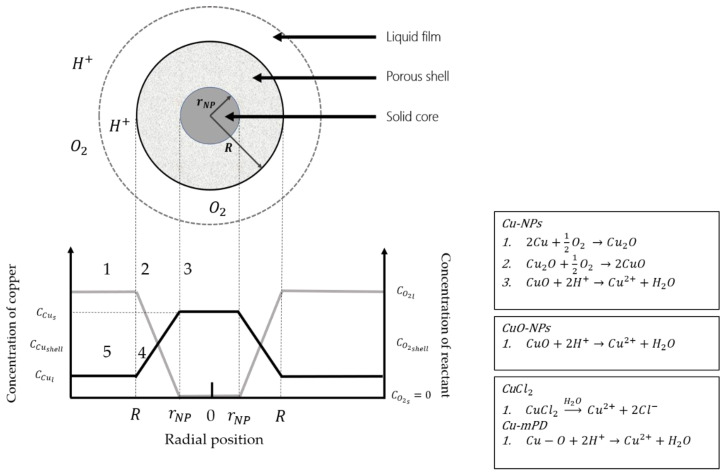
Scheme of the Shrinking Core Model over a spherical Cu-NP of initial radius R immersed on water. $r_{NP}$ corresponds to the remaining solid core at a given time. The concentration of copper ($C_{cu}$, black line) and reactant (in this case, oxygen and expressed as $C_{O_{2}}$, gray line) over the radial position are represented below and corresponds to a diffusion through the liquid phase and porous shell [40]. The chemical reactions that take place for the three copper species in study are also included.

A model based on the shrinking core model (SCM) could be useful to determine the control step in NPs dissolution and the effect of the polyamide layer when they are incorporated in the TFC-RO membrane. The SCM is a heterogeneous kinetics model that considers particles with spherical morphology and describes the depletion of the particles in time due to a chemical reaction that takes place in the fluid in which it is immersed [40]. Moreover, while the particle reacts, it forms a soluble product and a porous layer called shell, which is formed around a solid core of the particles (see Figure 1). SCM has been widely used to study leaching reactions and solid-gas in different applications [41,42]. But, to our knowledge, this model has never been used either to describe the dissolution and depletion of metallic nanoparticles in an aqueous media nor in the case of nanoparticles incorporated in a polymeric matrix. The transport mechanism is explained in detail in Section 2.5.

Thus, this work establishes a mathematical and experimental methodology to understand the copper-based NPs dissolution of three copper species incorporated into TFC-RO membranes. They are Cu-NPs, CuO-NPs nanoparticles and Cu-Oligomer complexes formed in situ during the interfacial polymerization process of the membrane. This work evaluates the biocidal capacity of the three copper species into the membrane and measures the copper ions release kinetics for both copper-based NPs in suspension and modified TFC-RO membranes. In addition, a dissolution model based on the shrinking core model (SCM) is applied and evaluated for the determination of the rate limiting step in the dissolution process and for prediction and simulation of the ion release kinetics. Therefore, the model is fitted and validated with the experimental data, and the results are compared with the biocidal capacity of copper species to get more details about the toxic mechanism of copper-based NPs when incorporated in the TFC-RO membranes. 

## 2. Materials and Methods 

### 2.1. Materials

The polysulfone (PS) Udel P-3500 MB7 (in pellet form, molecular weigth 83,000 g/mol; Solvay Advanced Polymers, Brussels, Belgium), 1-methyl-2-pyrrolidinone (NMP, >99.5%, Sigma Aldrich, Darmstadt, Germany) and *N*,*N*-dimethylformamide (DMF, >99%, Sigma Aldrich) were used to fabricate the PS sample used as support. The PA layer was synthesized from *N*-methyl-1,2-phenylenediamine (mPD) monomers and trimesoyl chloride (TMC), obtained from Sigma Aldrich (Darmstadt, Germany). CuCl_2_ powder (99%), commercial Cu (<25 nm) and CuO (<50 nm) nanoparticles were obtained from Sigma Aldrich (Darmstadt, Germany). Finally, NaOH (>97%) and n-hexane (>95%) were obtained from Sigma Aldrich (Darmstadt, Germany).

### 2.2. Synthesis of Copper Modified TFC Membranes

#### 2.2.1. Production of PS Support

The PS support was fabricated using the phase-inversion method according to the previously reported synthesis [19,20,21,22]. A PS solution (15% weight) was prepared by dissolving a polysulfone pellet in DMF:NMP (4:1) mixture for 2 h at 65 °C. DMF was used as principal solvent and NMP was used as pore former agent [19,43]. Afterwards, the PS casting solution obtained was uniformly dispersed on a glass plate using a casting knife (BYK, Geretsried, Germany) with the knife gap at 200 µm. The PS support was then immersed in water as coagulation bath at 25 °C. This PS support was recovered from the coagulation bath after 1 min and washed thoroughly with distilled water to remove any residual solvent. 

#### 2.2.2. Synthesis of TFC Membranes

The TFC-RO membranes were synthesized by interfacial polymerization reaction of the aqueous phase of mPD and the organic phase of TMC according to the previous reported protocol [20,21]. The PS support was immersed horizontally in a 2 wt% mPD aqueous solution containing 0.05 wt% NaOH during 2 min. The excess solution was removed of the support using a delicate task wiper (Kimwipes). Next, the membrane was immersed horizontally in 0.2 wt% of TMC solution in hexane for 1 min. The membrane was subsequently cured in an air-circulation oven n (Thermo Fisher Scientific, Madrid, Spain) a 75 °C for 8 min. Finally, the resulting TFC membrane (PA/PS) was washed with distilled water and dried at room temperature for 24 h. 

The modified membranes were prepared by homogeneous dispersion of Cu-based nanoparticles (0.25 wt% in 100 mL of pure water) in mPD aqueous solution (2 wt%) containing 0.05 wt% sodium hydroxide (NaOH) using ultrasonic stirring. This procedure was realized to avoid the NPs agglomeration on PA layer of the membrane. Later, NPs-mPD solution was added on the porous PS support and it was kept in contact for 2 min to produce modified membranes with Cu and CuO. For the formation of the in situ Cu-mPD oligomer complex the solution CuCl_2_ (0.25 wt%) in the mPD was kept in contact for 2 h. The impregnated PS support was subsequently immersed in the 0.2 wt% TMC solution in hexane, cured, washed, and dried using the same procedure previously described. The membranes modified by Cu, CuO and the in situ Cu-mPD oligomer are referred as (PA-Cu)/PS, (PA-CuO)/PS, and (PA-CuMPD)/PS, respectively.

### 2.3. Bactericidal Test

The differences in the bactericidal effect of the copper species incorporated into the modified TFC-RO membranes was evaluated by colony forming unit method (CFU) using *Eschericihia coli* (*E. coli*), which performs as model gram-negative bacteria. A pre-inoculum was first prepared by cultured 100 μL of bacteria in Luria-Bertani Broth (LB) solution and incubated in a shaking incubator at 200 rpm and 37 °C overnight. In parallel, new Schott bottles with 1 L of LB medium were prepared with membranes of each type. An additional bottle containing a sample of an unmodified membrane (PA/PS) was prepared. Next, 600 nm Optical Density (OD) measurement was applied to the bacteria solution to assure maintaining the bacteria concentration equal for all the bottles, $1\times 10^{7}$ cel/mL approximately.

The bottles were inoculated and kept in agitation at 200 RPM and 30 °C for 48 h. Samples of 1 mL of solution were taken from the bottles at the same intervals used in the ion release test. Afterwards, the solutions containing bacteria were serially diluted with PBS. From the highest dilution, 50 μL of the solution was pipetted into LB agar plates and then spread over the entire surface. The agar plates were incubated at 37 °C overnight and colonies developed were observed for to estimate the number of viable E. coli remaining in the suspensions.

The biocidal capacity of copper is obtained from the CFU results, quantified by the growth inhibition rate indicator (*I*), which was calculated using the following equation:

(1) $$I\left(\%\right)=\frac{\mu_{C}-\mu_{B}}{\mu_{C}}\times 100$$

where $\mu_{C}$ corresponds to the growth rate of control and $\mu_{B}$ to the growth rate of the modified membranes [24].

### 2.4. Copper Ion Release Test

The biocidal capacity of the copper species in the modified membranes was evaluated. Accordingly, the dissolution of the different types of copper species under study and the changes in their dissolution process when incorporated into TFC-RO membrane were consequently evaluated as well. Each sample (copper in suspension or modified membrane) with a concentration of copper of 2.5 g/L was individually added into a Schott bottle with 1 L of distilled water to keep the same initial amount of copper used in the biocidal test. The bottles were sealed and kept in agitation at 200 RPM and 30 °C. Samples of 30 mL were directly taken from the bottles. Firstly, within a range of minutes, and later within days during 240 h. These samples were centrifuged for 10 min at 6000 RPM and then filtered, to separate the Cu^2+^ ions from the NPs. The total amount of copper ion was measured using atomic absorption (AAS, GBC model SensAA dual, Melbourne, Australia).

### 2.5. Copper Dissolution Model Development

In order to understand the differences in the dissolution process of the copper species and its impact in the toxic capacity to bacteria, the SCM is used to simulate and compare the copper ion release from the nanoparticles both in suspension and the modified RO membranes.

Firstly, it is necessary to determine the control step in the reaction to then fit the dissolution curves. There are 5 steps involved to control the reaction (see Figure 1): 1. Diffusion through the liquid film in which the particle is involved, 2. Diffusion of the reactant through the shell, 3. Reaction on the surface of the particle core to form the product and shell, 4. Diffusion of the soluble product of the reaction through the porous shell and 5. Diffusion of the product of the reaction through the liquid film to the fluid [40].

In this study, the copper nanoparticle dissolves in water to form cupric ions (Cu^2+^). On the one hand, Cu-based NPs can suffer oxidation, which forms a layer on the nanoparticle core that can alter their dissolution process [39,44,45]. In case of non-elemental copper types (CuO and Cu-mPD) the steps involved are 1, 3 and 5 (see Figure 1). On the other hand, polymeric materials and coatings can passivate the dissolution process for NPs [26]. Hence, it is possible to propose a common model for the nanoparticles in suspension and incorporated nanoparticles in TFC-RO membranes through SCM [46].

Due to the high reactivity of water in the dissolution process, diffusion through the liquid film and the porous layer is very fast, which means that the two first steps of SCM are negligible as limitation step [40,47]. Therefore, only the last 3 steps of SCM are possible. In order to get to know the control step in the dissolution process of copper nanoparticles, the derivations of the equations are made individually. Nanoparticles are assumed homogenous, all of them with radius equal to R (known). The equations that define the ion transport in each step are the following:

(2) $$F_{nr}=k_{s}{\left[Cu^{2+}\right]}_{s}4\pi r_{NP}^{2}$$

(3) $$M_{psh}=\frac{D_{e}}{\left(\frac{1}{r_{NP}}-\frac{1}{R}\right)}\left({\left[Cu^{2+}\right]}_{shell}-{\left[Cu^{2+}\right]}_{s}\right)4\pi$$

(4) $$M_{lf}=k_{l}\left({\left[Cu^{2+}\right]}_{l}-{\left[Cu^{2+}\right]}_{shell}\right)4\pi R^{2}$$

The demonstration of each equation has been described by Levenspiel (1999) [40]. In case of the velocity of the system is controlled by a mix of stages, the final model will be an overall equation incorporating each controlling stage. Equations (2)–(4) describes the third, fourth and fifth steps of the SCM, respectively. $F_{\mathrm{nr}}\;\left(\frac{mol}{s}\right)$ is the reaction rate in the nucleus core, $M_{psh}$ and $M_{lf}\;\left(\frac{mol}{s}\right)$ corresponds to the copper mass transferred by diffusion through the porous shell and the liquid film, respectively. $\left[Cu^{2+}\right]\left(\frac{mol}{m^{3}}\right)$ is the concentration of the cupric ions, which might be on the liquid film (${\left[Cu^{2+}\right]}_{l}$), in the porous layer (${\left[Cu^{2+}\right]}_{shell}$) or on the solid core surface (${\left[Cu^{2+}\right]}_{s}$); $r_{NP}\;\left(m.\right)$ corresponds to the nucleus radius and is time-depending (i.e., in t=0; $r_{NP}=R$). $k_{l}\;\left(\frac{m}{s}\right)$, $D_{e}\;\left(\frac{m^{2}}{s}\right)$ and $k_{s}\;\left(\frac{m}{s}\right)$ are the mass transfer coefficient in the liquid film, the effective diffusion through the porous shell layer and the chemical reaction constant, respectively. In order to correlate these equations with the ion release, it is necessary to determine the copper nucleus consumption rate, so that:

(5) $$\frac{dN_{NP}}{dt}=\frac{d\left(\rho_{NP}V_{NP}N_{o}\right)}{dt}=\frac{N_{o}\rho_{NP}d\left(\frac{4}{3}\pi r_{NP}^{3}\right)}{dt}=\rho_{NP}\times 4\pi r_{NP}^{2}\frac{dr_{NP}}{dt}\times N_{o}$$

where $N_{NP}$ is the molar flux coming from the nanoparticles (mol/s); $\rho_{NP}\;\left(\frac{kg}{m^{3}}\right)$ is the density of the nanoparticles, considered as constant with time. $N_{o}$ is the apparent number of nanoparticles in the medium (in suspension or in the RO membrane). The conversion of the reaction (denoted as $X_{NP}$) is expressed as follows:

(6) $$X_{NP}=1-\frac{volume\;of\;unreacted\;core}{volumen\;of\;the\;unreacted\;particle}=\frac{\frac{4}{3}\pi r_{NP}^{3}}{\frac{4}{3}\pi R^{3}}={\left(\frac{r_{NP}}{R}\right)}^{3}$$

For each rate limiting step, a relation between the conversion and the copper concentration and design parameters of the nanoparticles is derived as follows:

(7) $$\frac{t}{\tau_{s}}=1-{\left(1-X_{NP}\right)}^{\frac{1}{3}}$$

(8) $$\frac{t}{\tau_{shell}}=1-3{\left(1-X_{NP}\right)}^{\frac{2}{3}}+2\left(1-X_{NP}\right)$$

(9) $$\frac{t}{\tau_{l}}=X_{NP}$$

where Equation (7) correspond to the chemical reaction on the surface of the particle core, working as a limiting step rate with the time of depletion of the nanoparticle equal to $\tau_{s}=\frac{\rho_{NP}R}{k_{s}{\left[Cu^{2+}\right]}_{s}}\;\left(s.\right)$, the total time taken for complete reaction and conversion of the nanoparticle (i.e., $r_{NP}=0$). Equation (8) is for porous shell control as a rate limiting step with time complete of conversion $\tau_{shell}=\frac{\rho_{NP}R^{2}}{6D_{e}{\left[Cu^{2+}\right]}_{s}}\;\left(s.\right)$. Finally, Equation (9) stands for liquid film control as a rate limiting step, where $\tau_{l}=\frac{\rho_{NP}R}{3k_{l}{\left[Cu^{2+}\right]}_{l}}\;\left(s.\right)$.

Particularly, the chemical reaction occurring on the surface of the NP core can be described by a kinetic equation (Equation (2)) which depends on the kinetic constant *k_s_*. This constant includes all chemical reactions that occur on the NP surface. In case of Cu-NPs, the *k_s_* involves the kinetic constants of Cu_2_O formation, CuO generation, and finally the copper dissolution. In case of non-elemental copper NPs, *k_s_* constant involves only the copper dissolution. Therefore, the *k_s_* must be different for each NP assessed.

To determine which step is the rate limiting in the copper dissolution process, the SCM Methodology is used [40]. This methodology is based on a linearization of the experimental data obtained from the dissolution curves in the ion release experiment (see Section 2.3). The linearization is made from three conversion equations presented above (Equations (6)–(8)) and follows the style y=1/τ×x with x=t and y the right-side of the control equations. The linearization with the best adjusted correlation coefficient (Adjusted R-square) is considered the limiting step rate. The mass transport equation of the corresponding case is used to fit the curves using minimization of the mean squared error (MSE) with dissolution constants as fitting parameters. The main assumptions of the model are the following:

- The particle size of NPs used is the average value reported by suppliers.
- The SCM assumes that particles are perfectly spherical.
- The SCM assumes a regular shape of the particle surface.
- The TFC membrane is assumed as part of the porous layer in the SCM.

## 3. Results and Discussion

### 3.1. Biocidal Effect of Copper Species on Modified TFC Membrane

**Figure 2:**
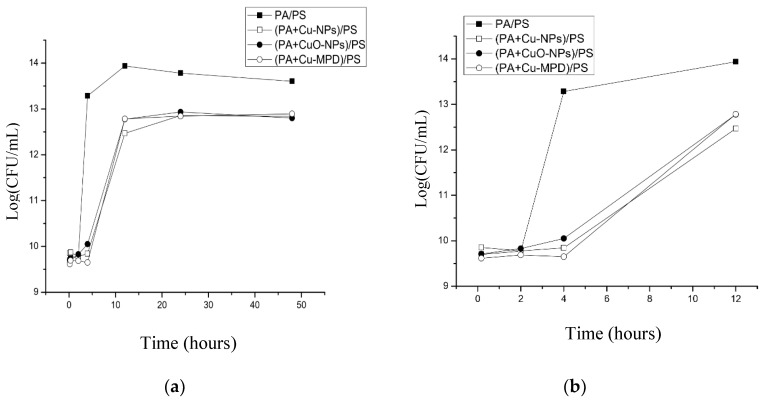
Bacteria growth curves in presence of pristine membrane (PA/PS) and copper-modified membranes, (**a**) *E. coli* CFU growth curves for the different experiments. (**b**) *E. coli* CFU growth experiments in the first 12 h.

Figure 2a shows the *E. coli* growth curves in the presence of the copper-modified TFC membranes and the pristine membrane (PA/PS) using CFU method.

The growth curves exhibit a standard behavior. In the first hours there is a lag phase where the bacteria are adapting to the media. After that, they reach the exponential phase, where there is a maximum growth. As the available nutrients become depleted, the bacteria reach a stationary phase where the death rate is equal to the growth rate. Finally, a death phase occurs, when the nutrients are completely consumed, which cannot be observed in these experiments [48].

The growth curves of bacteria cultured with the modified membranes by incorporation of copper-based NPs showed a significant reduction of the total bacteria compared with the pristine membrane (PA/PS) due to the presence of copper [23,49]. The number of total bacteria at 4 h of experiment is higher on the pristine membrane in comparison to the modified membranes (see Figure 2b), showing a strong impact on the bacterial growth. Thus, in the presence of a copper- modified membrane, bacteria are not able to leave the lag phase, in comparison to the PA/PS membrane that at 4 h is in exponential growth. This effect can be associated with the early release of toxic copper ions from the modified membranes [50]. After that time, although the bacteria in the presence of copper reach the exponential phase of growth, their steady state achieves a smaller number of cells compared to the control. Although a similar level of growth is observed, no significant differences between the copper species are noted during the experiment.

### 3.2. Copper Ion Release Kinetics

**Figure 3:**
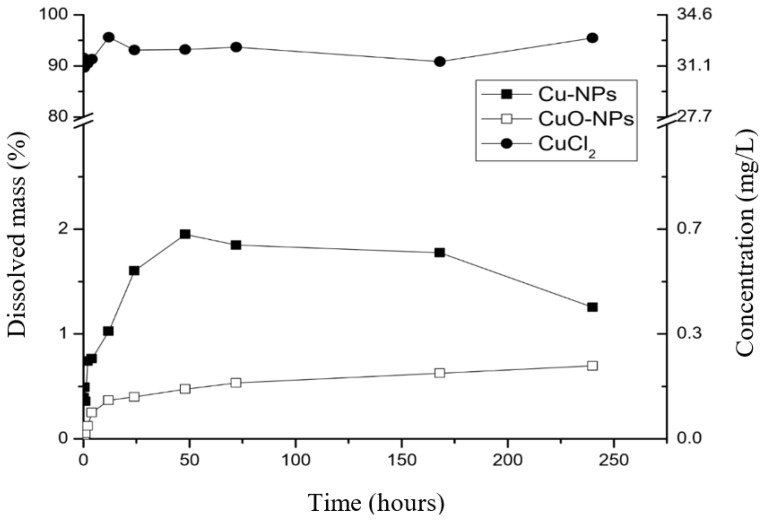
Copper ion release kinetics for Cu-NPs, CuO-NPs and CuCl_2_ salt.

**Figure 4:**
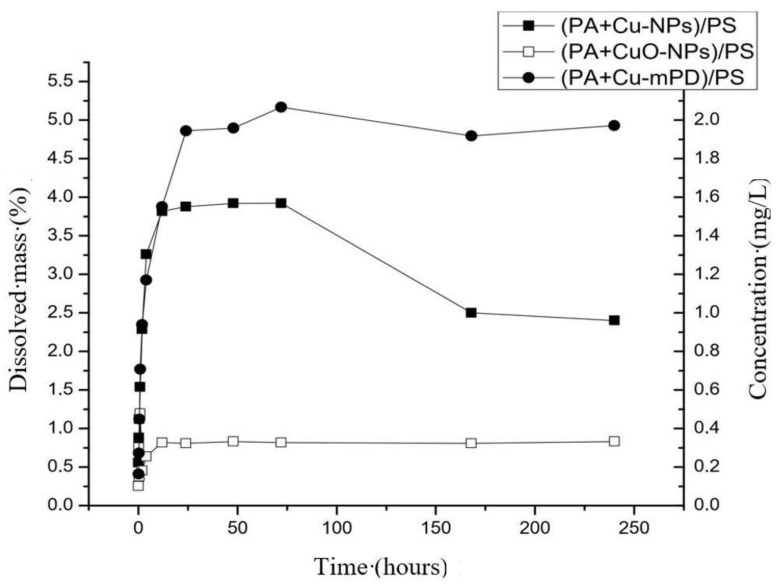
Copper ion release kinetics for TFC-RO membrane modified by incorporation of copper-based NPs.

Copper ion release from copper nanoparticles in suspension and the modified TFC-RO membranes were studied (Figure 3 and Figure 4, respectively).

Copper-based NPs dissolution curves in suspension were presented as the dissolved mass percentage with respect to the initial mass and copper ions concentration (Figure 3). Both nanoparticles (Cu-NPs and CuO-NPs) have a first-order dissolution curve, reaching their maximum dissolution near the 48 h of experiment. The percentage of dissolved copper from CuO-NPs is lower than to the Cu-NPs with respect to the total initial mass. CuO-NPs keeps continuous dissolution but in a minor rate, a fact associated with the oxide-state of copper in CuO-NPs which limits its dissolution capacity compared to Cu-NPs, and due to the less soluble surface of CuO. Midander et al. [49], reported that elemental Cu-NPs have a rapid initial dissolution that decrease in prolonged exposition due to the formation of an oxide layer that inhibits the dissolution.

On the other hand, a decrease in the copper ions concentration for Cu-NPs is noted over 150 h. The Cu-NPs needs oxygen to be oxidized and then dissolved (see chemical reactions in Figure 1), opposite to the behavior of the other copper types, since they only are dissolved. Furthermore, it is important to remark that the tests were performed in a sealed vessel. Therefore, in the Cu-NPs tests, the concentration of dissolved oxygen could have decreased with respect to the time, limiting the copper oxidation. In this case, the copper dissolution could have been stopped and then the chemical equilibrium could change, promoting the further precipitation of cupric ions to CuO. Similar results were obtained by Cioffi et al. [26] in their study of copper nanoparticles embedded in polymeric composites, but no explanation was given to the phenomenon. In order to propose a precipitation mechanism and explain this phenomenon, further studies must include the effect of dissolved oxygen, the oxidation-reduction potential or developing an electrochemical-based study. A transmission electron microscopy (TEM) to the nanoparticles previous and after the experiments could also give evidence of the copper oxide species present on the NPs surface.

Finally, the dissolution of the copper salt (CuCl_2_) used for the formation of the copper-oligomer complex into the membrane was also studied, showing a complete dissolution in a short period (<10 min).

Figure 4 shows the copper dissolution curves from the membranes modified by incorporating Cu-NPs, CuO-NPs and copper-oligomer complex during the interfacial polymerization process, defined as (PA+Cu-NPs)/PS, (PA+CuO-NPs)/PS and (PA+Cu-mPD)/PS, respectively. The copper dissolution curves of modified membranes showed a similar behavior with respect to kinetic dissolution of nanoparticles in suspension, with first-order kinetics (see Figure 3). Membrane modified with oligomer-complex Cu-mPD reach the highest concentration, followed by Cu-NPs and finally CuO-NPs. These different behaviors can be associated with the different interactions between the polyamide layer of TFC membranes with each incorporated copper species, impacting in their dissolution process.

For instance, Rodriguez et al. [22] studied the synthesis of modified membranes with copper salt added in situ during the interfacial polymerization process. In this case, they proposed a mechanism of formation of copper oligomer-complex in coordination with atoms of the polyamide layer of the membrane. In this regard, the addition of CuCl_2_ salt to the monomer solution (mPD) promote that one fraction of the mPD reacts with copper ions to produce a Cu-mPD oligomer complex, through chelation of amino groups of the monomer with Cu^2+^. Subsequently, this Cu-mPD oligomer complex is self-assembled with the PA layer during interfacial polymerization process by means of an interaction between copper ions of the oligomer complex and oxygen atoms of the carbonyl groups of the nascent PA layer, forming the modified membrane. On the contrary, other previous studies have remarked that during the incorporation of Cu-NPs and CuO-NPs into the TFC membranes, these NPs could be embedded into the PA layer [21,28], limiting the release of copper ions with respect to the capacity observed in (PA+Cu-mPD)/PS.

A comparison between modified membranes and the respective nanoparticle was evaluated. The maximum dissolution concentration reached by (PA+Cu-NPs)/PS membrane is higher compared to Cu-NPs in suspension (see Figure 3 and Figure 4). This fact can be related with the reaction of Cu-NPs in suspension with hydrogen ions to form cupric ions (Cu^2+^). Thus, an oxide layer is generated around the copper core that alters its dissolution process due to passivation [39,44,45,49]. In modified membrane ((PA+Cu-NPs)/PA), the PA layer could reduce the oxidation of the Cu-NPs reducing the CuO formation and increasing the concentration of ion release. These effects have been reported in previous works [39,44,45]. Kanninen et al. [44], studied the influence of ligands in the stability and oxidation of small copper nanoparticles (<5 nm) stabilized with capping agents. It was found that thiols and oleic acid made a ligand-exchange with the original capping agent, diminishing the oxidation and reaching greater dissolution of the nanoparticles. In addition, in this case a concentration decrease was observed. Similar to results of Figure 3, the oxygen availability could explain this phenomenon. As was mentioned earlier, the copper dissolution in Cu-NPs is promoted, firstly, by oxygen and secondly by hydrogen ion (Figure 1). In this context, the oxygen content can limit and control the copper ion release [49]. Thus, future works may consider studying aspects such as dissolved oxygen concentration, pH, and Eh conditions.

On the other hand, this effect is not observed on the (PA+CuO-NPs)/PS membrane, due to high chemical stability of the CuO-NPs [29,49], maintaining a similar dissolution concentration for both modified membrane and nanoparticles in suspension.

Finally, ion release capacity of the (PA+Cu-mPD)/PSf membrane showed a dissolution kinetics of the first order, similar to the one observed in both Cu and CuO-NPs. This behavior differs from the copper salt, which has a high solubility (see Figure 3). The change in the dissolution control observed on this modified membrane is associated with the formation of the copper oligomer-complex, which imposed a control over the copper ion release in the membrane [22,50].

### 3.3. Rate Limiting Step Determination

**Figure 5:**
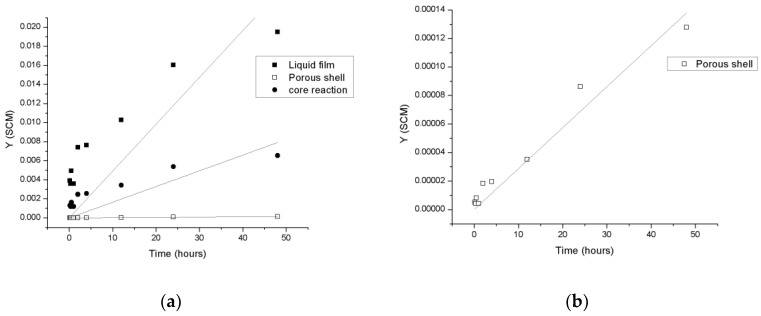
Rate limiting linearization for step determination of SCM-Model. The data used for this graphic is from Cu-NPs dissolution curve. Y corresponds to the right side of the step control equations (Equations (7)–(9)) which is adjusted considering the conversion of nanoparticles into copper ions. (**a**) includes the three step linearization curves and (**b**) only of porous shell linearization curve.

Figure 5 shows the linear approximation of the experimental data obtained from the dissolution curves of ions of the elemental-copper nanoparticles in suspension (see Figure 3). The data correspond to the transient phase and are fitted by the SCM Methodology to determine control step through the Equations (6)–(8). The rate limiting step is determined by the best correlation coefficient of the linearization ($R^{2}$), as specified in the materials and methods section. This methodology is repeated for the three copper species in this study. Table 1 shows the correlation coefficients of the data with the SCM linearization for Cu and CuO-NPs in suspension.

Table 1 shows that the two nanoparticles (Cu-NPs and CuO-NPs) have a great correlation coefficient for the porous shell, which functions as the control step, with a $R^{2}$ of 0.97 and 0.91, respectively (both are highlighted in bold), which confirms the applicability of the SCM-Model to the dissolution process of copper-based NPs. According to the dissolution mechanism proposed in Figure 1, the expected controlling step in case of CuO-NPs should be the liquid film or core reaction. In this case, the aggregation of NPs in suspension could be affected the contact area or particle shape, fitting the experimental results closer with a porous shell mechanism. The copper salt was excluded from this calculus because its dissolution process is not adjustable through the SCM.

Table 2 shows the linearization of dissolution experimental data for the copper-modified TFC membranes, using the same SCM linearization method. The membranes (PA+Cu-NPs)/PS and (PA+CuO-NPs)/PS keep the porous shell as control step such as the Cu-NPs and CuO-NPs in suspension, confirming that the SCM-Model can be used when the NPs dissolution is limited by a polymeric layer (highlighted in bold). In general, the active layer of the TFC-RO membrane imposes a diffusional control over the reaction, acting as the porous shell of the SCM [26]. This is confirmed by our modeling results in Table 2, where for the (PA+Cu-mPD)/PSf, the best R-square value corresponds to the porous shell model (highlighted in bold), outcome that is directly associated with the formation of complex Cu-mPD.

The fitting of the SCM model and each controlling step equation can be affected by the assumptions of the model described in Section 2.5.

The main advantage of the SCM is a possibility to identify the controlling stage of the process, allowing for the simplification of the complex process of a heterogonous system, given the possibility to use only one equation to predict the overall velocity of the process, neglecting the contribution of the rest of stages involved (in terms of velocity contribution). In this case, the results (Table 1 and Table 2) demonstrate that the diffusion in the porous shell is the controlling stage of the process, determining that the copper ion release can be predicted only using the equation related to this stage (Equation (3)). In addition, the future efforts to improve this process should be focused on the understanding of the generation and control of the porous shell, including shape, area, porosity and tortuosity.

### 3.4. SCM Fitting

**Figure 6:**
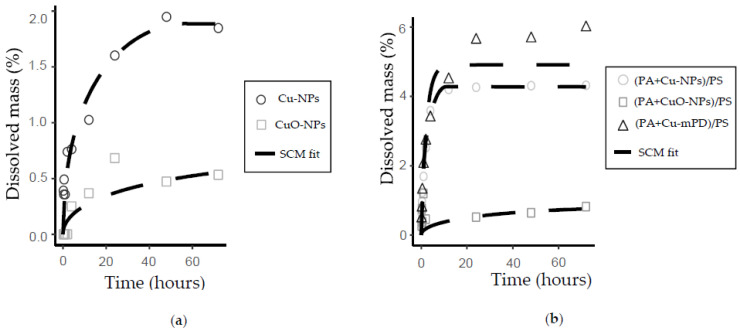
Copper release kinetics; the black dashed line shows the SCM model fit. (**a**) Copper in suspension; (**b**) Copper in the modified TFC-RO membrane.

With the determination of the rate limiting step, the copper ion release experimental data was fitted through the SCM using minimization of the MSE. Figure 6 shows the SCM fit to experimental dissolution curves of copper-based NPs in suspension and incorporated in the TFC-RO membranes (Figure 6a,b respectively), using Equation (7) of the porous shell as rate limiting step, according to the linearization results of Table 1 and Table 2. The fitting parameters used were the effective diffusion $D_{e}$ and the apparent number of nanoparticles $N_{o}$. For the latter, the reason to use it as a fitting parameter is that the nanoparticles are commercial, and, even the average diameter is known, its size distribution is unknown. Thus, the model can capture possible differences in the nanoparticles’ size that can alter their dissolution capacity.

The nanoparticles in suspension exhibit a greater fit with respect to experimental data as can be observed in Figure 6a. The values of the fit parameters and the summary of the correlation coefficients to the NPs in suspension are presented in Table 3. Correlation coefficients of 0.95 and 0.96 were obtained for Cu-NPs and CuO-NPs, respectively. The apparent number of Cu and CuO NPs are lower than those expected with respect to the initial mass and the average size of the particles used, which can be associated with a decrease of the surface area due to agglomeration of NPs in the media [38]. The fit corresponds to a first-order kinetics, where the time and shape of the transient phase depends on $D_{e}$ value, which is around $10^{-11}$ for Cu-NPs and $10^{-10}$ for CuO-NPs. Effective diffusion through a metallic solid or oxides is in the range from $10^{-10}$ to $10^{-13}$ $m^{2}/s$, which, according to the value of the fit, confirms the existence of the oxide layer around of the NPs and the porous shell as the control step [51,52].

Figure 6b shows the dissolution curves of modified membranes. The curves’ fit reach a good adjustment with experimental data in all cases, with correlation coefficients ranging from 0.91 to 0.98. In the case of (PA+Cu-mPD)/PSf membrane, there is not an inclusion of nanoparticles, but an in situ formation of the copper-oligomer complex. In this regard, a modification of SCM was realized. Nerst-Brunner equation [53,54], was used for fitting of the oligomer-complex, considering the porous shell diffusion as the rate limiting step based on the results in Table 2. The equation changes the constant value in Equation (2) to $\frac{D_{e}\times S}{\delta \times V}$, where S corresponds to the available area for dissolution, V is the volume of solution where the reaction occurs, δ is the porous shell thickness. This constant maintains the effective diffusion as fitting parameters, but the apparent number of nanoparticles changes following the solubility of cupric ions in water (referred as ${\left[Cu^{2+}\right]}_{S}$) which is generated by the oligomer-complex formed in situ. These coefficients and the fitted parameters for all Cu-based NPs incorporated in the TFC-RO membranes are summarized in Table 4. The estimated number of NPs for both Cu-NPs and CuO-NPs was very similar to the values obtained in the SCM fit for NPs in suspension, which was expected because the same mass of NPs was added in each case.

The values of $D_{e}$ of the modified membranes were determined. These are higher than those obtained for Cu-NPs and CuO-NPs in suspension. These differences could be attributed to the good dispersion and low agglomeration of NPs into the membrane, which is controlled during the synthesis process of the modified membranes, as has been optimized in our previous report [20,21,22,28]. The synthesis procedure could favor the shape, contact area and good dispersion of the NPs contained in the TFC-RO membrane to promote the effective diffusion. On contrary, in suspensions the agglomeration of NPs may be high and uncontrolled, limiting this diffusion. Furthermore, the thickness of the TFC membrane is slightly higher than the NPs particle size [20,21,28], favoring the proximity between NPs and the liquid phase. These phenomena could promote an effective diffusion coefficient higher than the obtained in the suspension tests.

Moreover, in the modified membrane this parameter is in the same order of magnitude for the three copper species (~$10^{-9})$. However, differences are observed among the modifications (Cu-mPD > Cu-NPs > CuO-NPs). The differences in the effective diffusion parameter between copper species incorporated in the TFC-RO membranes induce changes in the stationary states of copper dissolution. According to this, the values in the De parameter keeps the tendency of the stationary state results (Cu-mPD > Cu-NPs > CuO-NPs). These differences in the effective diffusion parameter in modified membranes may be associated with the possible changes in the interaction between the PA layer of the RO membranes and the copper-based NPs. As mentioned before, only the Cu-mPD proved the existence of a coordination between the copper and the PA layer [22]. Nevertheless, another type of coordination between Cu-NPs and CuO-NPs with the TFC-RO membrane could have an impact on this effective diffusion.

### 3.5. Ion Release Capacity and Biocidal Effect Comparison

In order to compare the toxic capacity of the copper species, the biocidal capacity was measured via the determination of the inhibition rate estimator (I), which corresponds to the reduction of the slope in the exponential phase with respect to the control. This is estimated using Equation (1). The results of the inhibition rate are presented in Table 5.

All the copper species in the modified RO membranes reach a value of I superior to 50%. This means that bacteria growth at half the speed in the presence of any copper species compared to control.

Thereby, the inhibition rate (*I*) reached by the copper species is similar, despite the fact that their stationary state of copper ions dissolution differs (see Figure 4), which was associated to the differences in the diffusion parameter of the SCM, as it was described above. These facts indicate that another factor in addition to the diffusion parameter and its impact in the ions dissolution could also influence on the toxicity mechanism of copper-modified TFC membranes upon reaching these similar inhibition rates.

An explanation could be associated with the copper-mediate ROS production capacity of the evaluated copper species. As mentioned before, ROS production is one of the main causes for copper toxicity and their production levels can vary depending on the type of copper. For instance, according to previous reports, CuO dissolution is a key factor to trigger ROS damage. Thus, CuO-NPs can induce formation of reactive oxygen species such as superoxide anions and hydrogen peroxide at very low sub toxic levels of dissolved copper (0.1 mg Cu/L) [55]. Similar results have been obtained for the toxic effect on bacteria, algae and human cells [16,26,32,56,57]. On the other hand, the differences in toxicity mechanism for copper-based NPs such as Cu-NPs and CuO-NPs compared to copper salts were studied by Kaweeterawat et al. They found that both NPs exhibit great ROS production, even at low concentrations of dissolved copper, compared to copper salts. However, in the case of Cu-NPs, interaction of the nanoparticles with the membrane of bacteria is key to ensure ROS-induced toxicity [34]. Accordingly, the incorporation of Cu-NPs in TFC-RO membranes could diminish ROS production due to the reduced direct contact with bacteria. Kaweeterawat also found that copper salts, although they were completely dissolved in short periods of time, cannot reach greater levels of ROS production as Cu-NPs and CuO-NPs.

Based on this and the results obtained in this work, although Cu-mPD reached the highest level of ion concentration, its ROS production could be limited compared to Cu-NPs and CuO-NPs. Therefore, the similar inhibition rate reached by copper species incorporated in the TFC-RO membrane could be determined by an equilibrium between the two previous mentioned factors: (a) the dissolution curve shape, defined by the effective diffusion parameter which is associated with the interactions between copper and PA layer, and (b) the ROS-mediate production which depends on the copper type. However, this last effect should be quantified. Accordingly, a new contribution that allows estimating the ROS production of each modified membrane is expected.

## 4. Conclusions

The SCM model was applied and evaluated to predict and simulate the ion release kinetics of three copper species incorporated into TFC-RO membranes: Cu-NPs, CuO-NPs and Cu-Oligomer complexes formed in situ during the polymerization process.

The linearization of the experimental dates by the SCM method showed that the diffusion though porous shell is the control step for all cases, both copper-based NPs in suspension and for the modified TFC-RO membranes with them, showing a diffusional control over the dissolution reactions with correlation coefficient $R^{2}$ around of 0.95. The diffusional control in the Cu and CuO NPs in suspension is associated with the formation of an oxide layer coating the nanoparticles. However, a minor but persistent ion release for the CuO-NPs is observed. On the other hand, when copper-based NPs are incorporated in the TFC-RO membranes, the PA layer impose the diffusional control over the reaction.

All modified membranes show a high biocidal capacity associated with the presence of copper. Modified membrane with Cu-Oligomer showed the highest released copper ions concentration (~5% of initial mass), followed by Cu-NPs (~4%) and CuO-NPs (<1%). However, a reduction over the 50% in the growth rate was obtained in all cases. In spite of the difference in ion release capacities, no significant statistical difference in biocidal capacity between membranes modified with the different copper species was observed. These facts can be due to the equilibrium between factors such as (a) the dissolution curve of copper ions, which is defined by the effective diffusion parameter and is dependent on the interactions between the copper type and the PA layer in the TFC-RO membranes and, (b) the ROS-mediate production which is more favorable for some species, such as CuO-NPs. Thus, these factors define the toxic capacity of the copper modified membranes and could enhance the selection of copper type and charge to reach an optimum antibiofouling RO membrane.

Regarding the above, this study is a first approximation to fully understand the toxic pathways of copper-modified TFC membranes. Future research must be focused on the estimation of ROS production of each modified membrane in order to better quantify their influence on the toxicity mechanism of copper-modified TFC membranes, as well as to understand the mechanism of copper ion release by the Cu-NPs, particularly the effect of dissolved oxygen. In addition, the implementation of the model developed in this work is an overall model of a reverse osmosis process, including the steady-state condition and hydrodynamic aspects, in order to estimate the copper ion profile with respect to the time, with perspectives to predict the life of the modified membrane in terms of anti-biofouling effect.

## 5. Patents

National patent CL201601310, registration number 58616.

## Figures and Tables

**Table 1:** Linear fit values of step limiting in SCM for nanoparticles in suspension. The limiting step and its correlation coefficient its highlighted in the table. The standard error of the linearization is also included.

Copper Specie	Step	Adjusted R-Square	Standard Error
Cu-NPs	Liquid film	0.774	$8.74\times 10^{-5}$
	**Porous shell**	**0.969**	$1.70\times 10^{-7}$
	Core reaction	0.775	$2.91\times 10^{-5}$
CuO-NPs	Liquid film	0.746	$6.29\times 10^{-6}$
	**Porous shell**	**0.907**	$7.36\times 10^{-9}$
	Core reaction	0.746	$2.09\times 10^{-6}$

**Table 2:** Linear fit values of step limiting in SCM for copper species incorporated in the TFC-RO membrane. The limiting step and its correlation coefficient its highlighted in the table. The standard error of the linearization is also included.

Copper Specie	Step	Asjusted R-Square	Standard Error
(PA+Cu-NPs)/PS	Liquid film	0.902	$1.25\times 10^{-4}$
	**Porous shell**	**0.999**	$1.18\times 10^{-6}$
	Core reaction	0.904	$4.18\times 10^{-4}$
(PA+CuO-NPs)/PS	Liquid film	0.669	$3.62\times 10^{-5}$
	**Porous shell**	**0.891**	$4.71\times 10^{-8}$
	Core reaction	0.671	$1.21\times 10^{-5}$
(PA+Cu-mPD)/PS	Liquid film	0.864	$1.42\times 10^{-4}$
	**Porous shell**	**0.979**	$4.64\times 10^{-6}$
	Core reaction	0.865	$4.76\times 10^{-4}$

**Table 3:** Fit parameters and coefficient of determination for dissolution curve of nanoparticles in suspension using SCM.

Copper Specie	$R^{2}\;\mathrm{Fit}$	Parameter			
		$D_{e}\;\left[m^{2}/s\right]$	Confidence Intervals (α=0.05)	$N_{0}\;\left[n^{{}^{\circ}}\;part.\right]$	Confidence Intervals (α=0.05)
Cu-NPs	0.96	$2.39\times 10^{-11}$	$\pm 2.38\times 10^{-12}$	$1.17\times 10^{12}$	$\pm 4.68\times 10^{9}$
CuO-NPs	0.92	$8.00\times 10^{-10}$	$\pm 1.40\times 10^{-11}$	$9.95\times 10^{10}\;$	$\pm 8.81\times 10^{10}$

**Table 4:** Fit parameters and coefficient of determination for dissolution curve of copper modifications in the TFC-RO membrane using SCM.

Copper Specie	$R^{2}\;\mathrm{Fit}$	Parameter				
		$D_{e}\;\left[m^{2}/s\right]$	Confidence Intervals (α=0.05)	$N_{0}\;\left[n^{{}^{\circ}}\;part.\right]$	${\left[Cu^{2+}\right]}_{s}\;\left[mg.\right]$	Confidence Intervals (α=0.05)
(PA+Cu-NPs)/PS	0.98	$5.93\times 10^{-9}$	$\pm 2.52\times 10^{-10}$	$2.53\times 10^{12}$	-	$\pm 1.55\times 10^{9}$
(PA+CuO-NPs)/PS	0.91	$3.14\times 10^{-9}$	$\pm 6.81\times 10^{-11}$	$9.45\times 10^{10}$	-	$\pm 2.76\times 10^{9}$
(PA+Cu-mPD)/PS	0.98	$6.91\times 10^{-9}$	$\pm 4.21\times 10^{-10}$	-	2.49	±0.7221

**Table 5:** Inhibition rate indicator for copper-based NPs incorporated in the TFC-RO membranes.

Membrane	I(%)
(PA+0.25%Cu-NPs)/PS	56.02 ± 4.84
(PA+0.25%CuO-NPs)/PS	58.34 ± 4.75
(PA+0.25%Cu-MPD)/PS	54.07 ± 0.83
